# Increased plasma levels of endozepines, endogenous ligands of benzodiazepine receptors, during systemic inflammation: a prospective observational study

**DOI:** 10.1186/s13054-014-0633-7

**Published:** 2014-11-18

**Authors:** Thomas Clavier, Marie-Christine Tonon, Anne Foutel, Emmanuel Besnier, Antoine Lefevre-Scelles, Fabrice Morin, Pierrick Gandolfo, Jean-Jacques Tuech, Muriel Quillard, Benoit Veber, Bertrand Dureuil, Hélène Castel, Vincent Compère

**Affiliations:** Institut National de la Santé et de la Recherche Médicale (Inserm), U982, Place Emile Blondel, 76130 Mont-Saint-Aignan, France; Normandy University, Institute for Research and Innovation in Biomedicine (IRIB), Place Emile Blondel, 76130 Mont-Saint-Aignan, France; Rouen University, Laboratory of Neuronal and Neuroendocrine Differentiation and Communication, Place Emile Blondel, 76130 Mont-Saint-Aignan, France; Department of Anesthesiology and Critical Care, Rouen University Hospital, Rue de Germont, 76000 Rouen, France; Department of Digestive Surgery, Rouen University Hospital, Rue de Germont, 76000 Rouen, France; Department of Medical Biochemistry, Institute of Clinical Biology, Rouen University Hospital, 76000 Rouen, France

## Abstract

**Introduction:**

Recent work has shown that benzodiazepines interact with the immune system and exhibit anti-inflammatory effects. By using *in vitro* models, researchers in several studies have shown that the peptidergic endogenous ligands of benzodiazepine receptors, named *endozepines*, are involved in the immune response. All endozepines identified so far derive from diazepam-binding inhibitor (DBI), which generates several biologically active fragments. The aim of the present study was to measure plasma levels of DBI-like immunoreactivity (DBI-LI) in a rat model of sepsis and in patients with systemic inflammation from septic or non-septic origin.

**Methods:**

Cecal ligation and puncture (CLP) or sham surgery was performed in rats. Blood samples were taken from animals, patients hospitalized for digestive surgery with inflammatory diseases, and healthy volunteers. Measurements of plasma DBI-related peptides were carried out by radioimmunoassay in animal and human samples.

**Results:**

In the rats, CLP provoked an increase of plasma DBI-LI (+37%) 6 hours postsurgery. In humans, DBI-LI levels were significantly higher in the systemic inflammation group than in the healthy volunteer group (48.6 (32.7 to 77.7) pg/ml *versus* 11.1 (5.9 to 35.3) pg/ml, *P <* 0.001). We found a positive correlation between endozepine levels and Acute Physiology and Chronic Health Evaluation II score (*r*_s_ = 0.33 (0.026 to 0.58), *P* < 0.05) and tumor necrosis factor α levels (*r*_s_ = 0.43 (0.14 to 0.65), *P* < 0.01). The area under the receiver operating characteristic curve for endozepines was 0.842 (95% CI (0.717 to 0.966), *P* < 0.0001) for discriminating patients with inflammation from healthy volunteers.

**Conclusions:**

Endozepines might be involved in the inflammatory response in patients with systemic inflammation.

## Introduction

Benzodiazepines are currently the most widely used drugs for prolonged sedation in intensive care units (ICUs) [1]. Nevertheless, the results of studies performed in animal models and humans suggest that prolonged treatment with benzodiazepines could increase mortality related to sepsis [2,3]. Accordingly, beyond their sedative effects, benzodiazepines also seem to have anti-inflammatory effects due to their interaction with the innate immune [4].

Sedative and/or hypnotic effects of benzodiazepines are mediated through type A γ-aminobutyric acid (GABA_A_) receptors, a chloride channel mainly expressed in the central nervous system (CNS) that exhibits benzodiazepine binding sites, named *central-type benzodiazepine receptors* (CBR) [5]. In contrast to CBR, peripheral-type benzodiazepine receptors, now renamed *translocator protein* (TSPO), are mitochondrial receptors coupled to an anion channel, mostly—but not only—located in peripheral tissues [6]. Thus, although they likely bind the same benzodiazepine ligands, potentiation of the activity of GABA_A_ receptors by benzodiazepines relays synaptic inhibition in CNS, whereas TSPO, which is broadly expressed in various organs (heart, kidney, liver, lung, adrenal glands) and cells (platelets, lymphocytes, mononuclear cells, endothelium, vascular smooth muscle, mast cells), may play a role in immune function and inflammation regulation [7,8].

The term *endozepines* designates a family of neuropeptides originally isolated from the rat brain as endogenous ligands of benzodiazepine receptors [9]. All endozepines derive from the polypeptide diazepam-binding inhibitor (DBI), generating through proteolytic cleavage of several biologically active peptides, including the octadecaneuropeptide DBI_33–50_ (ODN) [10]. Endozepines exert some of their effects through classical benzodiazepine receptors (that are, CBR and TSPO). DBI and ODN, which are both able to interact with CBR and to exert the inverse effects of benzodiazepines, are considered inverse agonists of this receptor. DBI is also considered an endogenous TSPO agonist; for example, DBI stimulates mitochondrial steroidogenesis through TSPO activation [9-11]. Endozepines are widely distributed in tissues and organs, notably in immune tissues (lymphocytes and monocytes), and the results of *in vitro* studies suggest an involvement of endozepines in cellular immune responses [12,13]. In particular, endozepines have been shown to (*1*) enhance lipopolysaccharide (LPS)-induced expression of tumor necrosis factor α (TNF-α), interleukin 1β (IL-1β) and interleukin 6 (IL-6) [14,15], and (*2*) stimulate chemotaxis, superoxide anion production and phagocytosis in isolated human neutrophils [16]. The prevention of immunosuppressive effects of benzodiazepines by a specific TSPO antagonist suggests that the anti-inflammatory effect of benzodiazepines is, at least in part, relayed by these receptors and that endogenous ligands of TSPO may play a role in inflammatory pathophysiological processes [17].

The function of endozepines during inflammation and sepsis remains unknown and knowledge concerning its secretion in blood during systemic inflammatory responses is lacking. We hypothesized that endozepines could be enhanced during systemic inflammation and sepsis because of their interaction with immune response. Thus, this study focused on the measurement of plasma endozepine levels in septic rats and in patients during systemic inflammation.

## Material and methods

### Preclinical rodent protocol

Sprague–Dawley male rats (Charles River, Elbeuf, France) weighing 200 to 250 g were housed under a constant temperature (21°C) in a 14-hr/10-hr light/dark cycle. The protocol was approved by the North-West Regional Ethic Committee on Animal Experimentation in France (referral number ceean0406-01, approval number 01-04-06/03).

Sepsis was induced by cecal ligation and puncture (CLP) as previously described [18]. Rats were anesthetized with ketamine (80 mg/kg) and xylazine (10 mg/kg). A 2-cm midline incision was made; the cecum was ligated below the ileocecal valve and punctured twice with an 18-gauge needle; and squeezed. The cecum was then placed back into the abdomen, and the incision was sutured. For sham-operated control rats, the cecum was exposed but was not ligated or punctured. The rats were killed by decapitation 1, 2, 3, 4, 6, 12 and 24 hours after surgery (5 animals/group for each time slot); trunk blood was collected in dried tubes; and the serum was obtained by centrifugation. Samples were stored at −80°C until endozepine extraction and radioimmunoassay (RIA).

### Clinical human protocol

#### Population studied

This study was conducted at the Rouen University Hospital from December 2006 to December 2009. It had been approved by the institutional review board (North-West Research Ethics Committee, Rouen University Hospital, France, referral number 2005/017) and therefore was performed in accordance with the ethical standards laid down in the 1964 Declaration of Helsinki and its later amendments.

Patients admitted to the digestive surgery unit with systemic inflammation were included. Exclusion criteria for patients with inflammation and healthy volunteers were age under 18 years or patient under tutorship, imprisoned inmates, pregnancy, participation in another clinical study, patient refusal and treatment with benzodiazepines within 3 months prior to inclusion. Patients were included in two groups after they provided their informed consent to participate: (1) healthy volunteers from the clinical investigation center and (2) patients with nonseptic inflammation or sepsis as defined by the Society of Critical Care Medicine [19].

The data collected included demographic characteristics, length of hospitalization, Acute Physiology and Chronic Health Evaluation II (APACHE II) score, biological levels of inflammatory markers (C-reactive protein (CRP), procalcitonin (PCT), TNF-α, IL-1β and IL-6).

#### Sampling

Samples taken during the patient’s hospitalization were scheduled between 8:00 AM and 9:00 AM because of the possible existence of a circadian rhythm influencing the release of endozepines. Blood was centrifuged (10 minutes at 3,000 *g* and 4°C), and serum samples were stored at −80°C until endozepine extraction and RIA.

#### Endpoints

The primary endpoint was the comparison of plasma endozepine levels between patients with inflammation and healthy volunteers. Secondary endpoints included (1) comparison between plasma levels of endozepine and other markers of inflammation, (2) assessment of potential correlations between endozepine levels and APACHE II scores and markers of inflammation and (3) determination of an endozepine level threshold that would enable us to discriminate healthy volunteers from patients with inflammation.

### Radioimmunoassay of endozepines (diazepam-binding inhibitor–like immunoreactivity) in rat and human plasma

Serum samples (rat: 1 ml, human: 2 ml) were diluted with 0.1% trifluoracetic acid (vol/vol) and heated at 56°C for 20 minutes. After centrifugation (3,200 *g* for 20 minutes at 4°C), peptides contained in the surpernatant were concentrated on Sep-Pak C18 cartridges (Alltech Europe, Lokeren Belgium). Bound material was eluted with 50% (vol/vol) acetonitrile/water containing 0.1% trifluoracetic acid (vol/vol), and the solvent was evaporated by vacuum centrifugation (Savant SpeedVac Concentrator; Thermo Scientific, Hicksville, NY, USA). The dry samples were resuspended in phosphate buffer (0.1 M, pH 8) containing 0.1% Triton X-100, and the concentrations of DBI-LI were quantified by RIA using antisera raised against synthetic rat DBI_33–50_ (rDBI_33–50_, QATVGDVNTDRPGLLDLK) or synthetic human DBI_33–50_ (hDBI_33–50_, QATVGDINTERPGMLDFT) (PolyPeptide Group, Strasbourg, France) in rabbit, as previously described [20]. Briefly, [Tyr^0^]-rDBI_33–50_ and [Tyr^0^]-hDBI_33–50_ were iodinated using the chloramine-T procedure and purified on a Sep-Pak C_18_ cartridge. The final dilutions of the antisera were 1:30,000 (rDBI_33–50_ antibodies) and 1:15,000 (hDBI_33–50_ antibodies). The total amount of ^125^I[Tyr^0^]-rDBI_33–50_ and ^125^I[Tyr^0^]-hDBI_33–50_ was 6,000 counts per minute for each tube. After a 2-day incubation at 4°C, the antibody-bound DBI-related fraction was precipitated by adding bovine γ-globulins (1%, wt/vol, 100 μl) and polyethylene glycol 8000 (20%, wt/vol, 2 ml). After centrifugation, the pellet containing the bound fraction was counted in a gamma counter (LKB Wallac, Rockville, MD, USA). Antibodies against rDBI_33–50_ and hDBI_33–50_ (ODN), respectively, detected the rat or human form of DBI [21,22]. Thus, rDBI_33–50_ and hDBI_33–50_ antibodies were not strictly specific for ODN and likely revealed the presence of DBI-related species in plasma.

### Measurement of inflammatory markers in human plasma

IL-6 was measured with an immunochemiluminescence assay (IMMULITE 2500; Siemens Medical Systems, Los Angeles, CA, USA). TNF-α and IL-1β levels were measured by enzyme-linked immunosorbent assays: Human TNF-α Ready-SET-Go kit (eBioscience, San Diego, CA, USA) and Human IL-1β Quantikine kit (R&D Systems, Minneapolis, MN, USA).

### Statistical analysis

Our presently reported work is an observational pilot study in which we investigated, for the first time to our knowledge, plasma levels of endozepines in patients with inflammation. Therefore, we did not establish an *a priori* calculation of the number of subjects required, but the inclusion of patients was performed to achieve a statistical power greater than 90% for nonparametric tests.

Values presented in the text and figures are median with 25th to 75th interquartile range or average with standard deviation. The Mann–Whitney *U* test was applied to determine statistical differences (α risk of 5%, β risk of 20%). Correlation was tested using Spearman’s rank correlation coefficient (*r*_s_). Receiver operating characteristic (ROC) curve analysis, including area under the ROC curve (AUC), was used to determine an endozepine level threshold able to discriminate groups. Samples whose endozepine level was below the minimum detection threshold of the assay were excluded from the statistical analysis because of the impossibility of assessing the primary endpoint. Subjects with unmeasurable endozepine levels were compared to those with measurable levels by using Fisher’s exact test. Statistics were calculated using GraphPad Prism software (GraphPad Software, La Jolla, CA, USA).

## Results

### Preclinical evaluation of endozepines in rat plasma during sepsis

**Figure 1:**
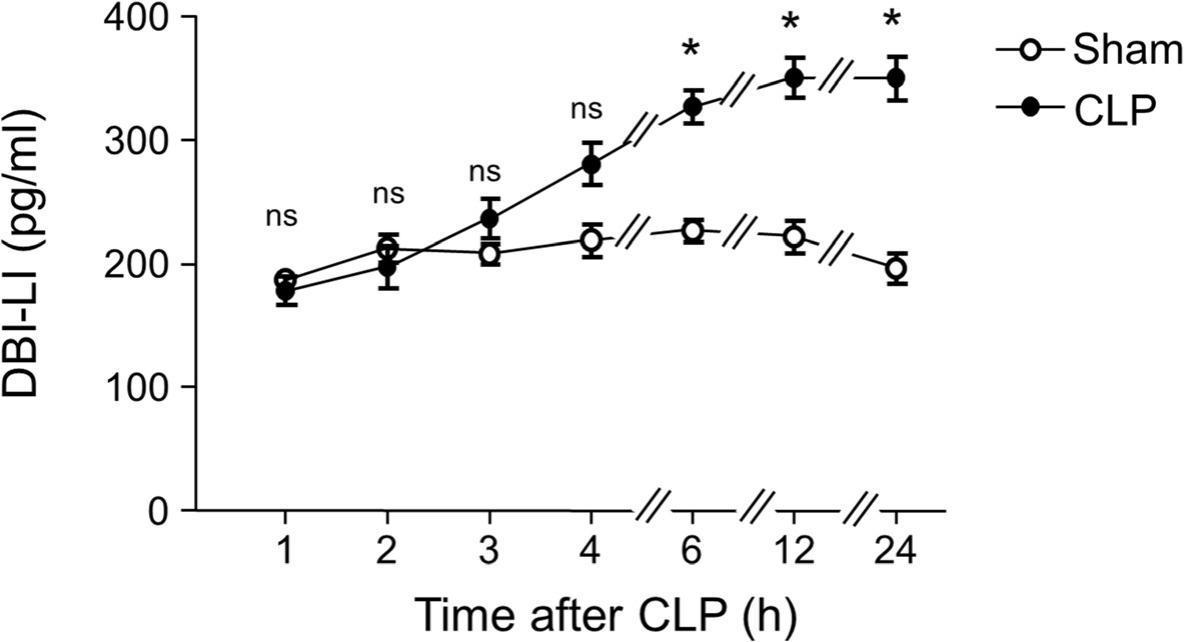
**Time course of the effect of cecal ligation and puncture on diazepam-binding inhibitor–like immunoreactivity in rat plasma.** Diazepam-binding inhibitor–like immunoreactivity (DBI-LI) was measured at the times indicated, after surgery, in the sera of sham-operated (SHAM, open circles) and cecal ligation and puncture (CLP, filled circles) animals (*n* = 5 animals/group for each time slot; each value represents the mean (± SEM) calculated from 5 animals). *P < 0.05 by Mann–Whitney *U* test; ns: not statistically different *vs.* SHAM.

Time-course experiments over a period of 24 hours revealed that CLP provoked a time-dependent increase in DBI-LI peptide concentrations in plasma. This increase was statistically significant after a 6-hour period (+37%; *P* < 0.05) and reached a plateau at 12 hours (Figure 1).

### Clinical evaluation of endozepines in plasma of patients

#### Epidemiologic characteristics

Sixty-seven subjects were included during the study period: forty-four in the group of patients with inflammation and twenty-three in the healthy volunteer group. There were no significant differences regarding age, weight or sex ratios between healthy volunteers and patients (Table 1). Patients with inflammation had appendicular peritonitis (*n* = 13), appendicular abscess (*n* = 3), cholangitis (*n* = 7), biliary peritonitis (*n* = 3), acute pancreatitis (*n* = 10), acute cholecystitis (*n* = 3) or sigmoid perforation (*n* = 5).

**Table 1:** **Epidemiologic characteristics, endozepine, biomarkers of inflammation and cytokine levels of healthy volunteers and patients with inflammation** ^**a**^

	**Healthy volunteers (** ***n*** **= 16)**	**Patients with inflammation (** ***n*** **= 44)**
**Age (yr)**	37.0 [29 to 45]	44 [28 to 60]^b^
**Sex,** ***n*** **(M/F)**	8/8^b^	23/21
**Weight (kg)**	61 [59.5 to 71]	71 [62 to 80]^b^
**APACHE II score**	NA	3 [2–6]
**Length of stay (days)**	NA	5 [4–8]
**Creatinine (μ** **mol/L)**	UM	72 [61 to 79]
**DBI-LI (pg/ml)**	11.1 [5.9 to 35.3]	48.6 [32.7 to 77.7]^c^
**PMN (×10** ^**3**^ **/ml)**	UM	9.2 [6.7 to 13]
**CRP (mg/L)**	UM	189 [132 to 279]
**PCT (μ** **g/L)**	UM	0.25 [0.16 to 1.4]
**TNF-α** **(pg/ml)**	6.1 [3.1 to 13.7]	4.2 [2.9 to 5.7]^b^
**IL-1β** **(pg/ml)**	UD	0.37 [0.12 to 0.69]
**IL-6 (pg/ml)**	UD	48.7 [17.6 to 79.7]

^a^The results are expressed as median and 25th to 75th interquartile range. ^b^Not statistically different from healthy volunteers; ^c^ *P* < 0.001 *vs* healthy volunteers. APACHE II, Acute Physiology and Chronic Health Evaluation II; CRP, C-reactive protein, DBI-LI, Diazepam-binding inhibitor–like immunoreactivity; IL, Interleukin; NA, Not applicable, PCT, Procalcitonin; PMN, Polymorphonuclear neutrophils; TNF-α, Tumor necrosis factor α; UM, Unmeasured; UD, Undetectable.

#### Endozepine, biomarkers of inflammation and creatinine levels in patients with inflammation and in healthy volunteers

Endozepine levels were not detected in seven samples taken from the healthy volunteer group. The number of patients with an unmeasurable level of endozepines was significantly higher in the healthy volunteer group than in the patient group (7 *vs* 0, *P* < 0.01). In the healthy volunteer group, there was no statistical difference between the “endozepine measurable” subgroup (*n* = 16) and the “endozepine undetectable” subgroup (*n* = 7) on the variables age, weight, sex ratio and plasma levels of TNF-α. The number of patients included in each group for statistical analysis and the distribution of endozepine levels provided a statistical power for nonparametric tests of 99%. The DBI-LI levels were significantly higher in the group of patients with inflammation than in the healthy volunteer group (48.6 [32.7 to 77.7] pg/ml *vs* 11.1 [5.9 to 35.3] pg/ml, *P* < 0.001) (Table 1). All healthy volunteers had undetectable levels of IL-1β and IL-6. There was no difference of TNF-α levels between groups (Table 1).

**Figure 2:**
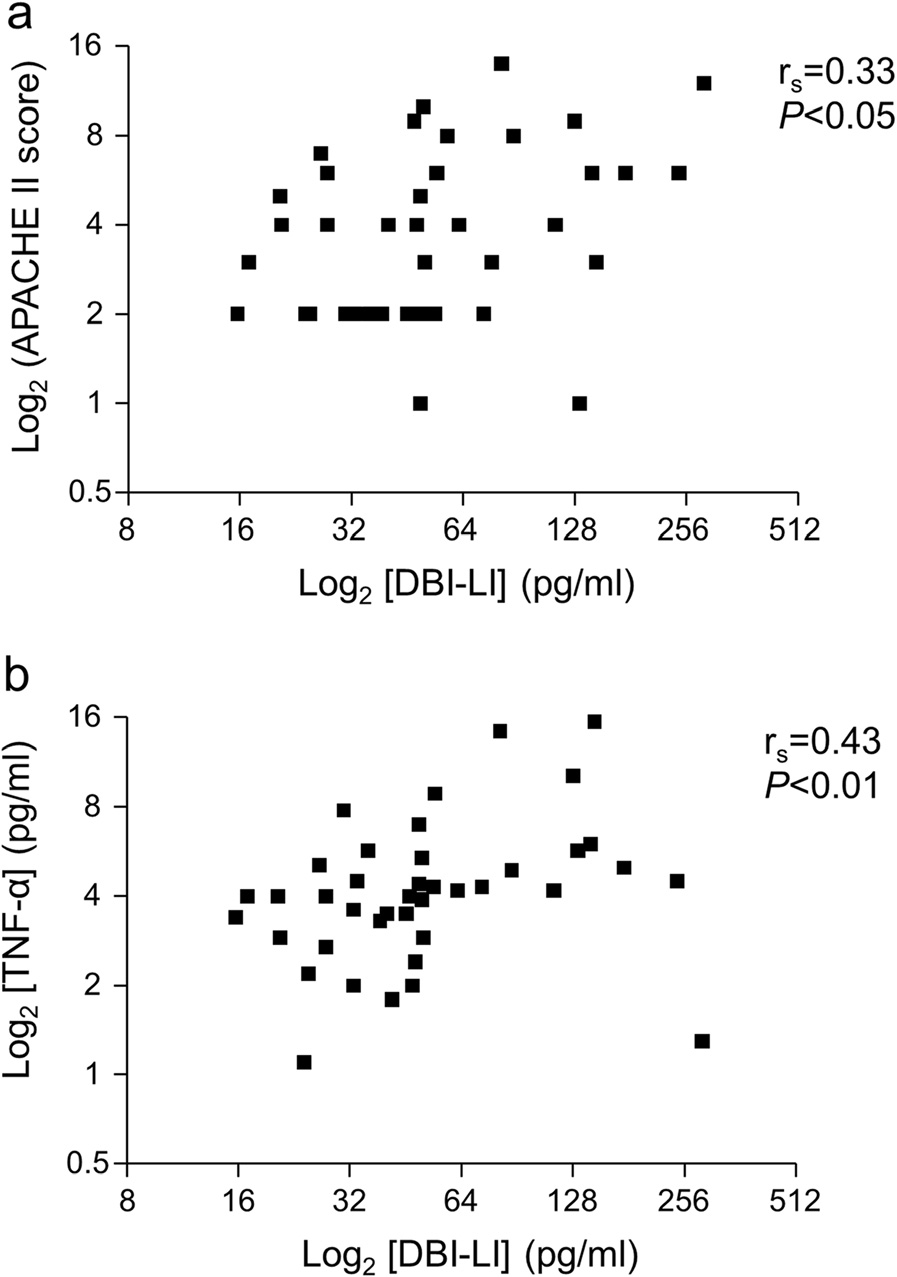
**Correlations between plasma endozepine concentrations and biomarkers of inflammation and Acute Physiology and Chronic Health Evaluation II scores in patients with inflammation.** Graphs depict the significant positive correlation between plasma endozepine levels and Acute Physiology and Chronic Health Evaluation II (APACHE II) scores in patients with inflammation **(a)** and between plasma endozepine levels and plasma tumor necrosis factor α (TNF-α) in patients with inflammation **(b)**. Correlation coefficients are Spearman’s coefficients (*r*_s_) with 95% confidence intervals. The results are presented as Log_2_ ratios. DBI-LI, Diazepam-binding inhibitor–like immunoreactivity.

When we studied patients with inflammation (*n* = 44), endozepine levels did not significantly correlate with IL-6, IL-1β, PCT and CRP levels (Table 2). However, there was a positive correlation between endozepine levels and APACHE II score (*r*_s_ = 0.33 [0.026 to 0.58], *P* < 0.05) (Figure 2a) and TNF-α levels (*r*_s_ = 0.43 [0.14 to 0.65], *P* < 0.01) (Figure 2b). When we studied patients with inflammation with an APACHE II score ≥ 6 (*n* = 12), we detected a positive correlation between endozepine and CRP levels (*r*_s_ = 0.78 [0.35 to 0.94], *P* < 0.01). We noticed that, in this subgroup, no correlation between endozepine levels and TNF-α, IL-6, IL-1β, PCT levels or APACHE II score was found (*data not shown*).

**Table 2:** **Correlation of endozepine levels with biomarkers of inflammation** ^**a**^

**Biomarker or APACHE II score**	**Spearman’s rank correlation**
IL-6	0.16 [−0.15 to 0.45]
IL-1β	−0.15 [−0.44 to 0.17]
TNF-α	0.43 [0.14 to 0.65]^b^
CRP	0.10 [−0.22 to 0.41]
PCT	0.089 [−0.26 to 0.42]
APACHE II score	0.33 [0.026 to 0.58]^c^

^a^CRP, C-reactive protein; IL, Interleukin; PCT, Procalcitonin; TNF-α, Tumor necrosis factor α. ^b^ *P* < 0.01. ^c^ *P* < 0.05.

#### Discriminative level of endozepine between patients with inflammation and healthy volunteers

**Figure 3:**
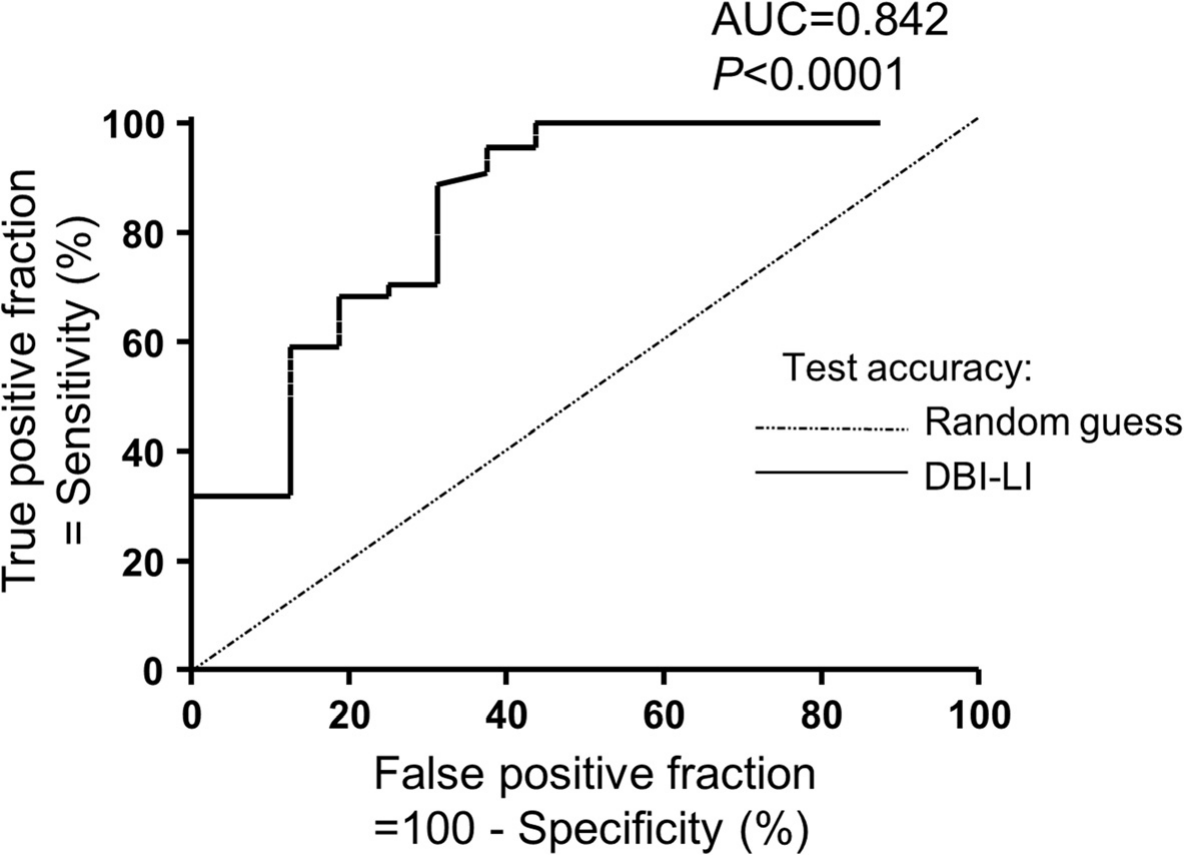
**Receiver operator characteristic curve generated with plasma levels of endozepines in patients with systemic inflammation and healthy volunteers.** The receiver operator characteristic (ROC) curve is the plot of the true positive rate (sensitivity) *vs* the false positive rate (100 − specificity) for different positivity thresholds (that is, different cutoff levels of endozepines). DBI-LI, Diazepam-binding inhibitor–like immunoreactivity. The calculated value of the area under the ROC curve (AUC) was 0.842 (superior to the random value of 0.5), indicating that DBI-LI plasma discriminated healthy volunteers from patients with systemic inflammation (*P* < 0.0001).

With regard to endozepines, the AUC for discriminating systemic patients with inflammation from healthy volunteers was 0.842 (95% CI, 0.717 to 0.966; *P* < 0.0001). With an endozepine cutoff value of 24.40 pg/ml, the sensitivity was 88.6% and the specificity was 68.8% (Figure 3).

## Discussion

The present data show, for the first time to our knowledge, that endozepine plasma levels are enhanced during systemic inflammation in both rats and humans. The study conducted in humans revealed that endozepine levels were increased in patients with inflammation compared with healthy volunteers.

Benzodiazepines are the most widely used drugs for prolonged sedation in the ICU [1]. *In vitro* studies suggested an immunosuppressive effect of benzodiazepines through an inhibition of cytokine secretion, dendritic cell antigen presentation or natural killer cell activity [4,23,24]. Non-benzodiazepine-based sedation could reduce mortality in septic patients in comparison with sedation using lorazepam [3]. However, this difference between benzodiazepine- and non-benzodiazepine-based sedation was not found in a recent meta-analysis of a general ICU population [25]. Thus, the impact of benzodiazepines on morbidity and mortality related to sepsis is still unclear. In this context, the characterization of plasma level variations of the endogenous ligands of benzodiazepine receptors, and raising their potential role during inflammation and sepsis, would provide new information on the potential side effects of benzodiazepine sedation in the ICU and would help to determine a potential use of specific CBR or TSPO ligands in inflammatory pathologies.

The results of the present study show that CLP induced a significant increase of endozepine plasma levels. These results led us to investigate the plasma concentration of endozepines in patients with inflammation and healthy volunteers. The concentrations of endozepines detected in plasma of healthy volunteers (11 [5.9 to 35.3] pg/ml in the present study) are of the same order as that measured by RIA (16 ± 5.3 pg/ml) in healthy controls of the same age in a previously published study, suggesting that the method of plasma endozepine detection used could be standardized within other clinical centers [26].

Tissues and signals responsible for the increase of endozepines in inflammatory subjects are currently unknown. Numerous tissues and organs express the *DBI* gene, and high levels of endozepines have been notably detected in basal conditions in human brain, liver, kidney, testis and adrenal glands [27,28]. Release of endozepines from rat hypothalamic explants has recently been demonstrated [29]. However, DBI mRNA expression in peripheral tissues and the release of endozepines from the intestine in rats suggest that the increase of circulating endozepines may result from peripheral organ release [27,28,30].

In our present study, measurement of cytokines in plasma showed very low or undetectable levels of TNF-α and IL-1β in all groups. This is consistent with the kinetics of TNF-α production. No IL-1β increase during sepsis was detected in other studies [31,32]. The positive correlation between endozepine and TNF-α plasma levels leads us to propose that the rise of plasma endozepines in inflammation could be either a consequence of overproduction of TNF-α or, conversely, a signal for cytokine production. In agreement with this hypothesis, it has been shown that the endozepine ODN potentiated the LPS-induced secretion of IL-6 by human monocytes [15]. In addition, it has been found that triakontatetraneuropeptide, a DBI-related peptide, and ODN potentiate the effects of LPS on the release of TNF-α and IL-1β by human monocytes and could thus maintain the inflammatory response [14,33]. The correlation between endozepines and TNF-α in patients with inflammation and endozepines and CRP in patients with inflammation with an APACHE II score ≥ 6 may also indicate that the increase of endozepines could be correlated with inflammation severity. However, given the low TNF-α concentration and the low overall inflammation severity in our patients, correlation with endozepine levels must be interpreted with caution. Although these data suggest a link between cytokines and endozepines, the connections between these two families of compounds remain to be elucidated.

The increase of endozepine levels in inflammation could have several consequences. In particular, TSPO has been found in many cells and tissues, including leukocytes such as macrophages and neutrophils, and also in steroidogenic tissues such as the adrenal gland [34]. These data suggest that TSPO could be a relevant target for endozepines during inflammation and sepsis. Besides this possible link between endozepines, TSPO and inflammation, it has been shown that ODN is a potent anorexigenic factor in rodents [35,36]. Thus, the increased levels of endozepines could be involved in anorexia related to acute inflammation. Moreover, in several *in vitro* studies, researchers have shown that endozepines inhibit glucose-stimulated release of insulin in rats, suggesting that an increase of endozepine levels could contribute to hyperglycemia induced by inflammation [37]. Future study of a possible relationship between endozepine, cortisol levels and glycemia during systemic inflammation to explore these hypotheses would be worthwhile.

The presently reported data indicate that endozepines are greatly increased in patients with inflammation, with an AUC of 0.842 to discriminate patients with inflammation *vs* healthy volunteers. Thus, the plasma endozepine level appears as efficient as CRP and PCT levels for the diagnosis of a biological systemic inflammatory response [38,39]. Nevertheless, the present work has several limitations. First, it is a single-center study including a limited number of patients, and only protocols including a larger number of patients in each group could allow identification of a stronger specific threshold. Second, the time of collection relative to the natural history of the disease was not standardized between patients. Such standardization would have been quite difficult, given the heterogeneity of the diseases included, but it will be necessary to standardize this parameter in a future clinical protocol including a large number of patients from various clinical centers. In addition, several data have not been identified at the time of sampling (for example, antibiotic therapy, diabetes, temperature), and it would have been be interesting to study the possible existence of a link between these factors and endozepine level. Third, the study design excluded patients with septic shock, and then all septic patients included comprised a non-ICU homogeneous population with abdominal infections with low APACHE II scores. Most of the patients with septic shock were sedated with benzodiazepines and, consequently, were excluded from our study. Interferences between exogenous benzodiazepines and endozepines were unknown; thus, treatment with benzodiazepines was initially an exclusion criterion. As a matter of fact, in a recent study, investigators showed that repetitive administration of diazepam stimulated DBI production in rats [40]. Together, it can be assumed that (*1*) measurement of plasma endozepines in this specific ICU population should be considered, (*2*) development of a nonseptic inflammatory model in rats should be suitable to highlight differences in levels of endozepines between septic and nonseptic inflammatory states and (*3*) variation of the CLP model severity (for example, by varying the size and/or number of cecal punctures) should be envisaged to study a potential link between the severity of sepsis and endozepine levels.

## Conclusions

Altogether, the present data show, for the first time to our knowledge, that plasma endozepines, endogenous ligands of benzodiazepine receptors, are enhanced in the inflammatory response in both rodents and humans. It would be interesting to study plasma levels of endozepines during severe sepsis and septic shock to determine whether endozepines could be proposed as a prognostic factor for serious infection, and also as a potential therapeutic target during septic and nonseptic inflammation. In this context, the next part of our work will consist of assessment of the endozepine course in plasma of septic patients during hospitalization.

## Key messages

- Endozepines, endogenous ligands of benzodiazepine receptors involved in cellular immune responses, are enhanced in the inflammatory response in humans.
- Tissues and signals responsible for this increase and its consequences in patients with inflammation are currently unknown and remain to be explored.

## Abbreviations

AUC: Area under the receiver operating characteristic curve
CBR: Central-type benzodiazepine receptor
CLP: Cecal ligation and puncture
CRP: C-reactive protein
DBI: Diazepam-binding inhibitor
DBI-LI: Diazepam-binding inhibitor–like immunoreactivity
ICU: Intensive care unit
IL-1β/6: Interleukin 1β/6
LPS: Lipopolysaccharide
ODN: Octadecaneuropeptide
PCT: Procalcitonin
RIA: Radioimmunoassay
ROC: Receiver operating characteristic
*r*_s_: Spearman’s rank correlation coefficient
TNF-α: Tumor necrosis factor α
TSPO: Translocator protein (peripheral-type benzodiazepine receptors)
